# Neutrophils to Lymphocyte Ratio: Earliest and Efficacious Markers of Sepsis

**DOI:** 10.7759/cureus.10851

**Published:** 2020-10-08

**Authors:** Fazal U Rehman, Asadullah Khan, Adil Aziz, Madiha Iqbal, Saad bin zafar Mahmood, Naureen Ali

**Affiliations:** 1 Department of Medicine, Aga Khan University Hospital, Karachi, PAK; 2 Department of Rheumatology, Fatima Memorial College of Medicine and Dentistry, Lahore, PAK; 3 Department of Nursing and Midwifery, Aga Khan University Hospital, Karachi, PAK

**Keywords:** neutrophil, lymphocyte ratio, c-reactive protein, procalcitonin, sepsis

## Abstract

Background

Neutrophil to lymphocyte ratio (NLR) can be easily calculated from the white cell differential count and is considered an auspicious marker for predicting different diseases, including sepsis. In this study, we aimed to compare the efficacy of NLR as a sepsis marker by comparing it with other markers of sepsis, such as C-reactive protein (CRP), procalcitonin, and the Sequential Organ Failure Assessment (SOFA) score.

Methods

A cross-sectional analytical study was conducted at the Aga Khan University Hospital from July 2019 to December 2019. A total of 168 patients who were admitted to the medicine department with a diagnosis of sepsis on arrival or during the hospital stay were enrolled. The neutrophil to lymphocyte ratio was calculated to form venous samples taken on admission and compared to the level of CRP, procalcitonin, culture reports, and the SOFA score as a predictor of sepsis.

Results

Out of 168 patients, 55.3% were male. The median age of the participants was 68.40 (interquartile range (IQR): 19.5) years in males and 64.0 (IQR: 18.0) in females. Procalcitonin was performed in 121 (72%) and CRP performed in 61 (36.3%) patients. The NLR showed significant associations with all the tested lab parameters of sepsis, such as CRP (p = 0.02), procalcitonin (p = 0.01), and SOFA score (p = 0.01). Values when analyzed according to culture-positive showed higher values in culture-positive samples but were not statistically significant.

Conclusion

Neutrophil to lymphocyte ratio is a cheap and rapidly available predictor of sepsis and has shown a significant correlation with other relatively expensive and non-rapidly existing markers of inflammation and sepsis. However, large prospective studies are needed to prove its real effectiveness as a marker of sepsis and its prognosis

## Introduction

Sepsis is a dysregulated inflammatory response to infection and manifests as a spectrum of illness that ranges clinically from bacteremia to severe sepsis to septic shock [1]. The Third International Consensus Definition for Sepsis and Septic Shock (Sepsis-3) defined sepsis as life-threatening organ dysfunction resulting from dysregulated host responses to infection and defined septic shock as a subset of sepsis in which underlying circulatory, cellular, and metabolic abnormalities are profound enough to substantially increase the risk of mortality [1]. Sepsis can originate from community-acquired infections or it can be hospital-acquired. The most common site of infection that leads to sepsis is the lung (62%), followed by the abdomen (20%), bloodstream (15%), and urinary tract 14% [2-3]. The clinical presentation of sepsis depends on the site of the infection. Common presentations include malaise and non-specific signs, such as fever (although hypothermia can be present too), tachycardia, tachypnea, or altered mental status. Arterial hypotension can be present, but its absence does not exclude sepsis [4]. Management is standardized globally but early diagnosis with aggressive resuscitation and infection control is emphasized [5]. With impressive results of the sepsis bundle of six hours of resuscitation, the Surviving Sepsis Campaign (SSC) group recently published newly defined “SSC Sepsis Bundles 2018”, advocating an hour-1 bundle, the first hour of resuscitation comprising of aggressive adequate fluid resuscitation, adequate antibiotics after cultures are acquired, and early use of vasopressors in case of fluid non-responsiveness [6-7].

Globally, high mortality is reported due to sepsis that lies from 25% to 30%, while locally it ranges from 36% to 76% [8-9]. This makes sepsis an “emergency” and requires urgent care, recognition, and management within hours [7-10]. NLR (neutrophils to lymphocytes ratio) has been studied in the pediatric population with sepsis and has gained importance in being effective markers of disease activity. However, the paucity of literature in the adult population and comparative studies with infectious markers, such as CRP (C-reactive protein test), procalcitonin, and the SOFA score (Sequential Organ Failure Assessment) needs further studies. However, in our study, we compared the levels of NLR with procalcitonin, CRP, and SOFA score as a marker of sepsis.

## Materials and methods

This cross-sectional analytical study was conducted at the Aga Khan University Hospital from July 2019 to December 2019. All the patients aged 17 years or above that were admitted to the Department of Medicine with a diagnosis of sepsis on arrival or during the hospital stay were included in the study. Patients with a history of blood loss, chronic liver disease, chronic kidney disease, and patients with autoimmune diseases that could have high inflammatory markers were excluded from the study. A sample of size of 168 patients was selected using a 10% level of significance, 90% confidence interval, and 5% bound of error with a 50% anticipated proportion of sepsis. A short survey was made that included demographic data, features of clinical examination, SOFA score, laboratory parameters, such as complete blood count (CBC), CRP, procalcitonin, and the primary source of sepsis determined by cultures, such as urine culture and sensitivity (CS), sputum CS, cerebrospinal fluid (CSF) culture, and blood cultures. The study was approved by the Aga Khan University Ethical Review Committee.

Data were analyzed using the Statistical Package for Social Sciences (SPSS), version 23 (IBM SPSS Statistics, Armonk, NY). A descriptive analysis was performed on all of the variables. Categorical variables were presented in the form of frequency and percentages. Continuous variables were presented in the form of the mean ± standard deviation having normal distribution, whereas the median interquartile range (IQR) were reported when there was asymmetric distribution. The normality of data was assessed by using Kolmogorov Smirnov's test. Association between demographic variables and gender were assessed by the Chi-square and Fisher's exact test. A p-value of ≤ 0.05 was considered significant.

## Results

One hundred and sixty-eight patients were included in the study, with male patients comprising 55.3% (n = 98) of the total. The median age of the participants was 68.40 (19.5 IQR) years in males and 64.0 (18.0 IQR) years in females. Table 1 elaborates on the baseline demographics and clinical parameters of sepsis in the subjects according to gender. The clinical parameters, such as heart rate, pulse, blood pressure, temperature, and respiratory rate, were comparable in both genders.

**Table 1:** Baseline Demographics and Clinical Characteristics of Participants According to Gender Values are either in mean +/- SD or n(%) or median (IQR) or n(%) for categorical variable *Median (IQR) for not symmetrically distributed quantitative variables **Mean ± SD for quantitative variable bpm: beats per minute; CNS: central nervous system; GCS: Glasgow Coma Scale; IQR: interquartile range; MAP: mean arterial pressure; SD: standard deviation; SOFA: Sequential Organ Failure Assessment; UTI: urinary tract infection

Characteristics	Total (n = 168) n(%)	Male (n = 98) n(%)	Female (n = 70) n(%)	P-value
Age*	66 (18)	68.40 (19.5)	64.0 (18.01)	0.12
Heart rate (bpm)**	98.20 ± 1.64	98.15 (2.3)	97.81 (2.3)	0.83
Blood pressure				
Systolic	118 (31)	115.0 (29)	119 (38)	0.13
Diastolic	70 (20)	70.0 (26.5)	70 (20)	0.08
MAP *	80 (25.50)	78.0 (23.50)	84.0 (27)	0.08
Respiratory Rate*	24 (9.0)	24 (8.5)	24 (10)	1.00
Temperature*	37.0 (1.0)	37.0 (1.0)	37.0 (1.0)	0.73
GCS*	15 (2)	15 (2)	15 (1)	0.77
SOFA score	6.0 (4.0)	6.0 (4.5)	5.0 (4.0)	0.58
Sepsis source				
Pneumonia	74 (44.0)	42 (56.8)	32 (43.2)	0.74
UTI	44 (26.2)	23 (52.3)	21 (47.7)	0.63
Intraabdominal source	9 (5.4)	5 (55.6)	4 (44.4)	0.99
CNS infection	8 (4.8)	4 (50)	4 (50)	0.75
Soft tissue/Skin	32 (19.0)	19 (59.4)	13 (40.6)	0.61

The median SOFA score was 6.0 (4.0 IQR), with a slightly higher but insignificant median value in males than females (6.0 with 4.5 IQR vs 5.0 with 4.0 IQR, respectively, p = 0.58). The most common source of sepsis occurring in 74 (44.0%) patients was pneumonia, followed by urinary tract infection (UTI) in 44 (26.2%), soft tissue/skin infection in 32 (19%), and intra-abdominal source in nine (5.4%), and CNS infection in eight (4.8%) patients. 

Table 2 elaborates on the laboratory parameters of the participants stratified according to gender. Procalcitonin was performed in 121 (72%) patients and CRP performed in 61 (36.3%) patients. The mean Hb was 10.73 ± 0.185 with significantly lower levels in the female vs. male gender (11.12 ± 0.26 vs 10.25 ± 0.24, respectively, with p = 0.02). The median TLC was found to be 13.9 (11.8). Except for platelets and total bilirubin showing significant association with gender (p-values 0.04 and 0.05, respectively), no other baseline lab showed any association with gender. Among markers of sepsis based on the SSC Sepsis Bundles 2018 [6], procalcitonin showed a significant association with the male gender (p = 0.04), while no other markers (CRP, NLR, and SOFA score) showed any association (Table 2). NLR showed significant associations with all the tested lab parameters of sepsis, such as CRP (p = 0.02), procalcitonin (p < 0.01), and SOFA score (p = 0.01) (Table 3). NLR values, when analyzed according to culture-positivty, showed higher values in culture-positive samples but were not statistically significant (Table 4). In addition, when analyzed for correlation with SOFA, only NLR and procalcitonin showed significant association (p = 0.01 each), while CRP and TLC did not show any significant association with the SOFA score (Table 5).

**Table 2:** Baseline Laboratory Characteristics of Participants According to Gender Values are either in mean +/- SD or n(%) or median (IQR) *Median (IQR) for not symmetrically distributed quantitative variables **Mean ± SD for quantitative variable CRP: C-reactive protein; IQR: interquartile range; NLR: neutrophil to lymphocyte ratio; PaO_2_/FiO_2_: partial pressure of oxygen/fraction of inspired oxygen; SD: standard deviation; TLC: total leukocyte count

Investigation	Total	Male	Female	P-value
Hemoglobin**	10.73 ± 0.185	11.12 ± 0.26	10.25 ± 0.24	0.02
TLC*	13.9 (11.8 IQR)	13.9 (10.80)	13.9 (11.50)	0.30
Neutrophils*	84.0 (13.2)	84.90 (10.75)	84.10 (13.90)	0.30
Lymphocytes*	8.0 (10.05 IQR)	8.0 (9.10)	8.3 (11.00)	0.55
NLR*	10.59 (13.5 IQR)	10.75 (13.07)	9.70 (13.60)	0.43
Platelets*	229 (161 IQR)	201 (117)	257 (202)	0.04
Creatinine*	1.80 (2.48 IQR)	1.80 (2.45)	1.70 (2.40)	0.59
Total bilirubin*	0.60 (0.80 IQR)	0.70 (0.50)	0.50 (0.90)	0.05
Lactate*	2.00 (1.50 IQR)	2.0 (1.45)	1.9 (1.55)	0.27
PaO_2_/FiO_2_*	364 (166 IQR)	361 184.6)	371 (153.0)	0.50
CRP*	105 (158.9 IQR)	98.25 (158.3)	107 (133.7)	0.70
Procalcitonin*	1.38 (12.35 IQR)	3.39 (14.05)	0.80 (6.30)	0.04

**Table 3:** Association of Neutrophil to Lymphocyte Ratio (NLR) with Markers of Sepsis CRP: C-reactive protein; SOFA: Sequential Organ Failure Assessment

Sepsis marker	Correlation	P-value
CRP	0.292	0.023
Procalcitonin	0.76	< 0.01
SOFA	0.214	0.012

**Table 4:** Neutrophil to Lymphocyte Ratio (NLR) Values According to Culture Positivity

Culture source	NLR value		P-value
	Culture-positive n(%)	Culture-negative n(%)	
Blood culture	12.25 (11.98)	9.56 (12.69)	0.10
Urine culture	11.2 (16.08)	10.39 (12.09)	0.75
Sputum culture	12.65 (17.56)	9.70 (12.93)	0.12
Pus culture	13.43 (8.37)	7.98 (13.98)	0.07

**Table 5:** Correlation of Markers of Sepsis with SOFA CRP: C-reactive protein; NLR: neutrophil to lymphocyte ratio; SOFA: Sequential Organ Failure Assessment; TLC: total leukocyte counts

	Correlation	P-value
TLC	0.01	0.87
NLR	0.21	0.01
CRP	0.06	0.9
Procalcitonin	0.2	0.01

## Discussion

The management of sepsis is quite challenging. The earlier and rapid response towards sepsis results in a higher chance of survival. This current study presents our experience of NLR as an earlier marker of sepsis at the first encounter that resembles with the baseline CBC. The study showed a significant association of NLR with multiple inflammatory markers and the sepsis score, namely, the SOFA score.

This study is consistent with previous studies considering NLR as a prognostic marker of sepsis [10-12]. These studies proposed NLR not only as a predictor of sepsis but also as a marker of disease severity and worse outcome. However, past literature not only proposed NLR to predict the severity of sepsis but it also serves as a prognostic marker of mortality [12-13]. Many studies have reported the usefulness of NLR as a sepsis marker regardless of the source of sepsis [14-17]. Meshaal et al., for example, proposed NLR in cases of infective endocarditis not only as a predictive marker of sepsis but also as an independent predictor of worse outcomes, such as hospital mortality [14]. De Jager et al., on the other hand, has reported NLR as an important marker of pneumonia, both as a diagnostic and prognostic measurement [15]. The ability of NLR as a predictive and prognostic marker of sepsis is not confined to the adult population but its feasibility has been witnessed in the pediatric and neonatal population [16-17].

The study also examined the association of NLR and other inflammatory markers with a SOFA score of sepsis severity. Among the different parameters studied, NLR and procalcitonin both showed a significant association with the SOFA score (p < 0.05). Previous research has also shown a significant association of NLR with SOFA and other severity sores of sepsis, although the association is not confined to a specific source of infection. For example, Velissaris et al. reported a significant association of NLR with SOFA and APACHE II (Acute Physiologic and Chronic Health Evaluation) scores [18]. Zhou et al. reported such a significant association in patients with sepsis-associated with acute pancreatitis, while Godinez-Vidal et al. reported the association in patients with appendicitis [19-20].

Our study compared NLR with other inflammatory markers as predictive and prognostic markers. Both CRP and procalcitonin were found to have significant associations with NLR. Similar results have been previously proposed by authors, such as Ljungström et al. and Xu et al., for CRP and by Merik et al. and Arif et al. for procalcitonin [21-24]. Most of the above-mentioned studies showed these associations to be of prognostic value as well [21, 23-24]. Zheng et al, however, reported CRP and procalcitonin to be better biomarkers than NLR for predicting sepsis [25]. Besides, the combination of NLR and CRP improved the diagnosis of early sepsis in study participants. 

NLR did not show any significant association with culture positivity in our study. The results are parallel with the study done by Loonen et al., who did not find any significant association [26]. In contrast, studies done by Naess et al. and Gozdas et al. found a significant association between NLR and blood culture positivity [27-28]. The differences observed could be attributed to variation in methodology, sample size, or study population. Treatment initiation at the arrival might be a contributing factor in decreasing the yield of culture positivity, making inflammatory parameters as an important marker. 

There are certain limitations of the study, as it is a retrospective cross-sectional study that makes the causal relationships difficult. We did not study the prognostic effects of NLR, such as mortality, morbidity, etc. Confounders, such as different treatments and comorbidities, were not controlled.

## Conclusions

NLR is a readily available significant inflammatory marker that is strongly associated with the onset and severity of sepsis and may be used as a predictor of sepsis in terms of an early marker, although future studies are required to report strong associations. Its effective use early in the course of sepsis, especially at Day 1 or at initial hours, will help to improve the management and outcomes of sepsis.
